# Molecular basis for histidine N3-specific methylation of actin H73 by SETD3

**DOI:** 10.1038/s41421-019-0135-5

**Published:** 2020-01-21

**Authors:** Yihui Zheng, Xingrun Zhang, Haitao Li

**Affiliations:** MOE Key Laboratory of Protein Sciences, Beijing Advanced Innovation Center for Structural Biology, Beijing Frontier Research Center for Biological Structure, Tsinghua-Peking Joint Center for Life Sciences, Department of Basic Medical Sciences, School of Medicine, Tsinghua University, Beijing, 100084 China

**Keywords:** Nanocrystallography, Methylation

Dear Editor,

As an extension of the central dogma, dynamic modifications of macromolecules play a key role in shaping cellular traits through modulating diverse cellular processes, such as gene regulation and signaling. In addition to lysine and arginine methylations that have been widely characterized in histone^1^ as well as nonhistone proteins^2^, histidine methylation stands out as another important type of protein/peptide methylation for biological regulation. Remarkably, histidine methylation could occur to N1 or N3 position of its imidazole ring, posing an interesting topic to understand the biology underlying position-specific methylation of histidine. Previously, we have revealed the structural basis for histidine N1-specific methylation by human CARNMT1^3^. Recently, SET domain-containing protein 3 (SETD3) was identified as an N3-specific histidine methyltransferase of β-actin at the highly conserved residue H73^4,5^. Functionally, N3 methylation of H73 is associated with actin polymerization and ATP hydrolysis^6–8^, highlighting a role of histidine methylation in regulating cell motility.

To decipher the molecular basis for actin H73 methylation by SETD3, we crystallized the ternary complex of SETD3_1–497_ bound to β-actin peptide (60–81) and sinefungin (SFG, a SAM analog), and determined the crystal structure at 2.7 Å (Fig. 1a; Supplementary Table S1). Based on the electron density map, we are able to build residues 20–494 of SETD3 and 66–81 of β-actin in the presence of SFG (Fig. 1b). Such a ternary structure well resembles a catalytic state before the methyl transfer from the SAM donor to the actin substrate. SETD3 is composed of a SET domain at its N-terminal lobe and a Rubisco LSMT substrate-binding domain at its C-terminal lobe (Fig. 1b; Supplementary Fig. S1). The SET domain consists of three discrete β-sheets (I: β1–β2; II: β3–β7–β6; III: β4–β5) and a bundle of α helices (α4–α13) inserted between β3 and β4, while the Rubisco LSMT domain contains seven α helices (α16–α22) and a pair of β-strands (β8–β9) (Supplementary Fig. S2a). The SET domain is responsible for SFG and β-actin peptide recognition, in which the SFG molecule is bound at a cleft formed by the β1–β2 hairpin and α14–β6; meanwhile the actin peptide is clamped by β4 and β6 with the H73 side chain pointing to SFG at the catalytic center (Fig. 1b; Supplementary Fig. S2a).

**Fig. 1:**
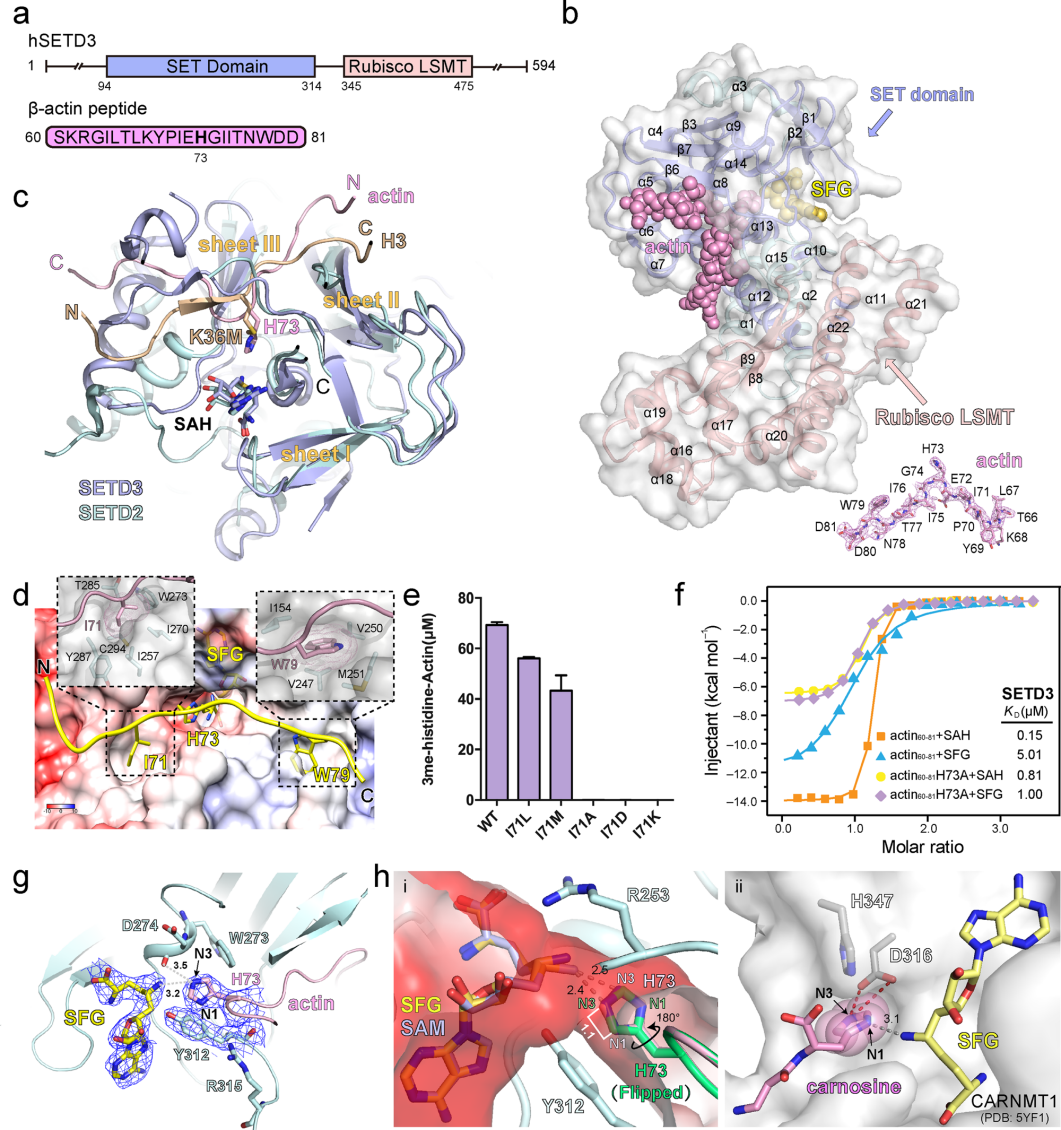
Biochemical and structural characterizations of actin H73 methylation by SETD3. **a** Domain architecture of human SETD3 and the sequence of actin peptide used for crystallization. **b** Overall structure of SETD3_20–494_ bound to actin peptide and SFG. The SET domain and Rubisco LSMT domains of SETD3 are color-coded as indicated. SETD3 is also shown in semitransparent surface view. Actin peptide and SFG are depicted as pink and yellow spheres, respectively. Fo–Fc omit map of the actin peptide (66–81) is contoured at 3.0 σ level. **c** Structural alignment of SETD3 and SETD2 catalytic SET domains. SETD3 and SETD2 are depicted as blue and cyan ribbons, respectively. The histone H3K36M peptide in the SETD2 complex is shown as a dark- yellow ribbon, and the actin peptide in the SETD3 complex is shown as a light-pink ribbon. Sheets I, II, and III: three core β-sheets conserved among SET domains. Note the overlap of H73 of actin and H3K36M at the catalytic center next to the SAH cofactor. **d** Registration of the actin peptide along the substrate-binding channel of SETD3. Close-up view highlights the hydrophobic pockets for I71 and W79. The protein surface of SETD3 is represented in electrostatic potential view expressed as a spectrum ranging from −10 kT/e (red) to +10 kT/e (blue). **e** Catalytic activities of wild-type (WT) and indicated I71 mutants quantified by LC–MS. Error bars represent standard deviation of three repeats. **f** Calorimetric titration-fitting curves of SETD3_20–494_ titrated with wild-type actin peptide or H73A mutant actin peptide in the presence of SFG or SAH, respectively. **g** Interaction details of the catalytic pocket. Electron densities of SFG, Y312 of SETD3, and H73 of actin peptide are shown as the Fo–Fc omit map contoured at 1.2 σ level. Numbers are distances in the unit of Å. **h** (i) “Head-to-head” engagement of SFG and actin H73 within the substrate channel of the catalytic center. The channel is colored as electrostatic potential surface as defined in panel **d**. A modeled SAM molecule is overlaid with SFG for analysis. A side-chain flipped histidine H73 is shown as overlaid green sticks. (ii) N1 position-specific methylation of carnosine by CARNMT1. Numbers are distances in the unit of Å.

SETD3 and SETD2 share a homologous catalytic SET domain. Structural alignment of the two SET domains revealed conserved overall substrate engagement mode, in which both peptide substrates are bound at the inner β-edge of sheet III with actin H73 and histone H3K36M (oncohistone H3 lysine to methionine mutation at site 36)^9^ being spatially overlapped and presented to the methyl donor (Fig. 1c; Supplementary Fig. S2b). Intriguingly, the actin and the histone peptides bind in opposite N-to-C orientations, and sequence motifs flanking actin H73 and H3K36 are recognized by distinct residues/surfaces of the two SET domains (Fig. 1c). These features determine the specificity of nonhistone versus histone substrates of SETD3 and SETD2.

In the complex structure, actin peptide 66–81 is docked into an electrostatically bipartite surface cleft of SETD3. The surface cleft is negative in the left and positive in the right, which well matches a basic-to-acidic residue distribution of actin peptide flanking H73 from the N to C terminus, and facilitates the binding orientation of the peptide (Fig. 1a, d). Interaction analysis revealed multiple sets of hydrogen bonding and ion pair interactions, as well as extensive hydrophobic contacts (Supplementary Fig. S3). Remarkably, actin H73 is inserted into a deep catalytic pocket at the center, which is flanked by two hydrophobic pockets accommodating I71 and W79, respectively (Fig. 1d). Specifically, I71 pocket is formed by residues I257, I270, W273, T285, Y287, and C294, while W79 pocket is formed by I154, V247, V250, and M251. Mutations of I71 into a small side chain or polar residues, such as I71A, I71D, and I71K, disrupted the methylation of actin H73 by SETD3; by contrast, I71L and I71M that retained a bulky and hydrophobic feature at position 71 only slightly weaken actin H73 methylation by SETD3 (Fig. 1e). These results stress an important role of the H73-flanking hydrophobic residues in registering actin H73 at the catalytic pocket of SETD3.

We measured a binding affinity of 0.15 μM by isothermal titration calorimetry (ITC) between actin peptide 60–81 and SETD3 in the presence of SAH, highlighting binding contributions of the observed protein–peptide interactions. Strikingly, the binding affinity dropped by >30-fold to 5.01 μM in the presence of SFG (Fig. 1f). Structural superimposition of our SETD3–SFG–actin ternary complex with the reported SETD3–SAH–actin structure^5,10^ revealed outer shifts of SFG and actin peptide backbone (Supplementary Fig. S4a), suggesting existence of repulsive tension between SFG and H73 of actin (Fig. 1g). In support, simple replacement of SAH with modeled SAM or SFG in the SETD3–SAH–actin structure revealed close contact between the “S-methyl/C-NH2” groups of SAM/SFG and the H73 side chain (Supplementary Fig. S4b). We next performed ITC titrations by using the H73A actin peptide that will not trigger steric clash at position 73 due to its smaller side chain, and measured binding constants of 0.81 μM and 1.00 μM in the presence of SAH and SFG, respectively (Fig. 1f; Supplementary Fig. S5). The loss of SFG sensitivity by using the H73A mutant peptide supports a role of steric repulsion in the observed >30-fold affinity drop. Both the methyl donor SAM and H73 of actin are confined in a narrow channel surrounded by residues including Y312 and R253 (Fig. 1hi). We reason that high-affinity binding of the actin substrate may compensate for a reduction of the activation energy during catalysis, and thus promotes the methyl transfer reaction through proximity and orientation effects^11^.

SETD3 is responsible for N3-position-specific methylation of actin H73, while CARNMT1 is responsible for N1 position-specific histidine methylation of carnosine. In the latter case, an acidic residue D316 forms a direct hydrogen bond to N3 of the histidine imidazole ring whose flipping is further constrained by residue H347, thus rendering only N1 available for methylation (Fig. 1hii)^3^. SETD3 adopts a different mechanism to achieve N3-position specificity. In this case, the SAM donor and the actin H73 are confined in a narrow channel, and they approach each other in a “head-to-head” manner (Fig. 1hi). In such a substrate engagement mode, no matter how the imidazole ring rotates, the N1 atom is ~1.1 Å away from N3; therefore, only N3 of actin H73 side chain is available for further methylation (Fig. 1hi).

In summary, we solved the co-crystal structure of SETD3_20–494_ bound to actin peptide (66–81) and SFG. Our studies revealed the structural basis for sequence motif and histidine N3-position-specific recognition and catalysis by SETD3. Through structural alignment of the SET domains of SETD3 and SETD2, we revealed that sequence motif is critical for peptidyl substrate selectivity. SETD3 cannot function as a histone methyltransferase owing to its unique binding surface features that favor nonhistone sequence motif for recognition. Different from the case of CARNMT1, the H73 side chain is precisely confined in a narrow substrate channel, and the resultant “head-to-head” engagement mode renders N3, but not N1 of actin H73 available for methylation. Actin participates in many important cellular processes, including muscle contraction, cell motility, cell division, and signaling. The precise installation of a methyl mark on H73 N3 of actin calls attention to an important regulatory mechanism centered on nonhistone and non-K/R methylations in modification biology.

The atomic coordinate of SETD3_20–494_ bound to actin peptide (66–81) and SFG has been deposited into the Protein Data Bank under accession code 6JAT.

## Supplementary information

Supplementary information

## Supplementary information

**Supplementary Information** accompanies the paper at (10.1038/s41421-019-0135-5).
