# Supplementary material for: Molecular basis for histidine N3-specific methylation of actin H73 by SETD3
Source: Cell Discov. 2020 Jan 21;6:3. doi: 10.1038/s41421-019-0135-5 (PMC6971037; doi:10.1038/s41421-019-0135-5)
Supplement: Supplementary file 1 — Supplementary information [file 41421_2019_135_MOESM1_ESM.pdf]

Supplementary information

Materials and methods

Protein expression and purification

Wild type and mutant full length human SETD3 (residue 1-497) was cloned into a pSUMOH10 vector (an in house modified SUMO tag vector based on pET28b) containing an N-terminal 10×His-SUMO tag. The recombinant $SETD3_{1-497}$ was overexpressed in *E. coli* BL21 (DE3) strain induced by 0.2 mM isopropyl β-D-thiogalactoside (IPTG) at 16°C overnight. Cells were harvested and suspended in lysis buffer containing 20 mM Tris, pH 8.0, 250 mM NaCl, 5% glycerol and 20 mM imidazole. After cell lysis and centrifugation, $SETD3_{1-497}$ was affinity-purified by a HisTrap column and eluted with a continuous gradient from 20 to 500 mM imidazole and then the 10×His-SUMO tag was cleaved by ULP1 overnight at 4°C which was further removed by reloading onto the HisTrap column. $SETD3_{1-497}$ was then purified by an anion exchange column (HiTrap Q HP, GE Healthcare) and finally polished by size-exclusion chromatography on a Superdex 200 10/300 GL column (GE Healthcare) in buffer containing 20 mM Tris, pH 8.0, 150 mM NaCl.

Crystallization, data Collection and structure determination

For crystallization of the $SETD3_{1-497}$-SFG-Actin peptide (60-81) ternary complex, the protein sample was prepared by directly mixing protein with Sinefungin (SFG, a SAM analogue) and actin peptide (60-81) in a molar ratio of 1:10:20 for overnight at 4°C before crystallization. The ternary complex was grown at 18°C by mixing equal volumes of the protein sample with the reservoir solution using the sitting-drop vapor diffusion method. The crystal was grown in

a reservoir solution containing 0.1 M Hepes-Na, pH 7.5, 0.2 M ammonium sulfate, 20%(w/v) PEG8000, 10%(v/v) 2-propanol.

The diffraction data set was collected at the beamline BL17U of the Shanghai Synchrotron Radiation Facility. All diffraction images were indexed, integrated and merged using HKL2000 software packages[1]. The structure was determined by molecular replacement using the MolRep program with the free structure of human SETD3 (PDB code: 3SMT) as the search model. Structural refinement was carried out using PHENIX[2], and iterative model building was performed with COOT[3]. Detailed structural refinement statistics are in Table S1. Structural figures were generated using the PYMOL (http:// www.pymol.org/) or Chimera (http://www.cgl.ucsf.edu/chimera) programs.

Isothermal titration calorimetry

All calorimetric experiments in this study were performed at 25°C using a MicroCal iTC200 instrument (GE Healthcare). Before ITC titration, SETD3$_{1-497}$ was incubated with SFG or SAH in a molar ratio of 1:10 at 4°C for 1 hour. The sample containing 200 μL of 50 μM protein was titrated with 17 successive injection of 1 mM peptide. Acquired calorimetric titration curves were analyzed using Origin 7.0 (OriginLab) using the “One Set of Binding Sites” fitting model.

***In vitro* methyltransferase assay**

The reaction was carried out in a 50 μL volume containing 10 μM purified wild type or mutant SETD3$_{1-497}$, 100 μM actin peptide, 500 μM SAM and 20 mM Tris, pH 8.0. The reaction was performed at 37°C for 10 hours and terminated by adding 0.1% TFA and then was subjected for LC-MS quantification.

Reference

1. Otwinowski, Z. & Minor, W. Processing of X-ray diffraction data collected in oscillation mode. *Methods Enzymol.* **276**, 307–326 (1997).

2. Adams, P. D. *et al.* PHENIX: a comprehensive Python-based system for macromolecular structure solution. *Acta Crystallogr. D Biol. Crystallogr.* **66**, 213–221 (2010).

3. Emsley, P. & Cowtan, K. Coot: model-building tools for molecular graphics. *Acta Crystallogr. D Biol. Crystallogr.* **60**, 2126–2132 (2004).

Supplementary Table S1

Data collection and refinement statistics

Crystal	SETD3-Actin-SFG
Data Collection	
Space group	$P2_12_12_1$
Unit Cell	
a, b, c (Å)	60.4, 131.3, 175.2
α, β, γ (°)	90, 90, 90
Resolution (Å)	50-2.70 (2.76-2.70)
No. of unique reflections	38,336 (2499)
R_{sym} (%)	16.2 (85.5)
R_{pim} /CC1/2 (%)	5.5 (29.7)/98.2 (83.9)
I/σ (I)	13.2 (1.8)
Completeness (%)	99.8 (99.8)
Redundancy	9.7 (9.2)
Refinement (F>0)	
Resolution (Å)	50-2.7
No. of unique reflections	38,266
R_{work}/R_{free} (%)	20.0/24.3
No. of non-H atoms	
Protein	7,694
Peptide	263
Water	84
SFG/Buffer ions	54/40
Average B-factors (Å^2)	
Protein	46.4
Peptide	45.7
Water	41.4
SFG/Buffer ions	34.0/56.1
RMSD bonds (Å)	0.005
RMSD angle (°)	0.705
PDB entry code	6JAT

Supplementary Table S2.

Sequence of β-actin peptide used in this paper

Peptide Name	Sequence
Actin (60-81)	SKRGILTLKYPIEHGIITNWDD
Actin (60-81) H73A	SKRGILTLKYPIEAGIITNWDD
Actin (66-78)	TLKYPIEHGIITN
Actin (66-78) I71L	TLKYPLEHGIITN
Actin (66-78) I71M	TLKYPMEHGIITN
Actin (66-78) I71A	TLKYPAEHGIITN
Actin (66-78) I71D	TLKYPDEHGIITN
Actin (66-78) I71K	TLKYPKEHGIITN

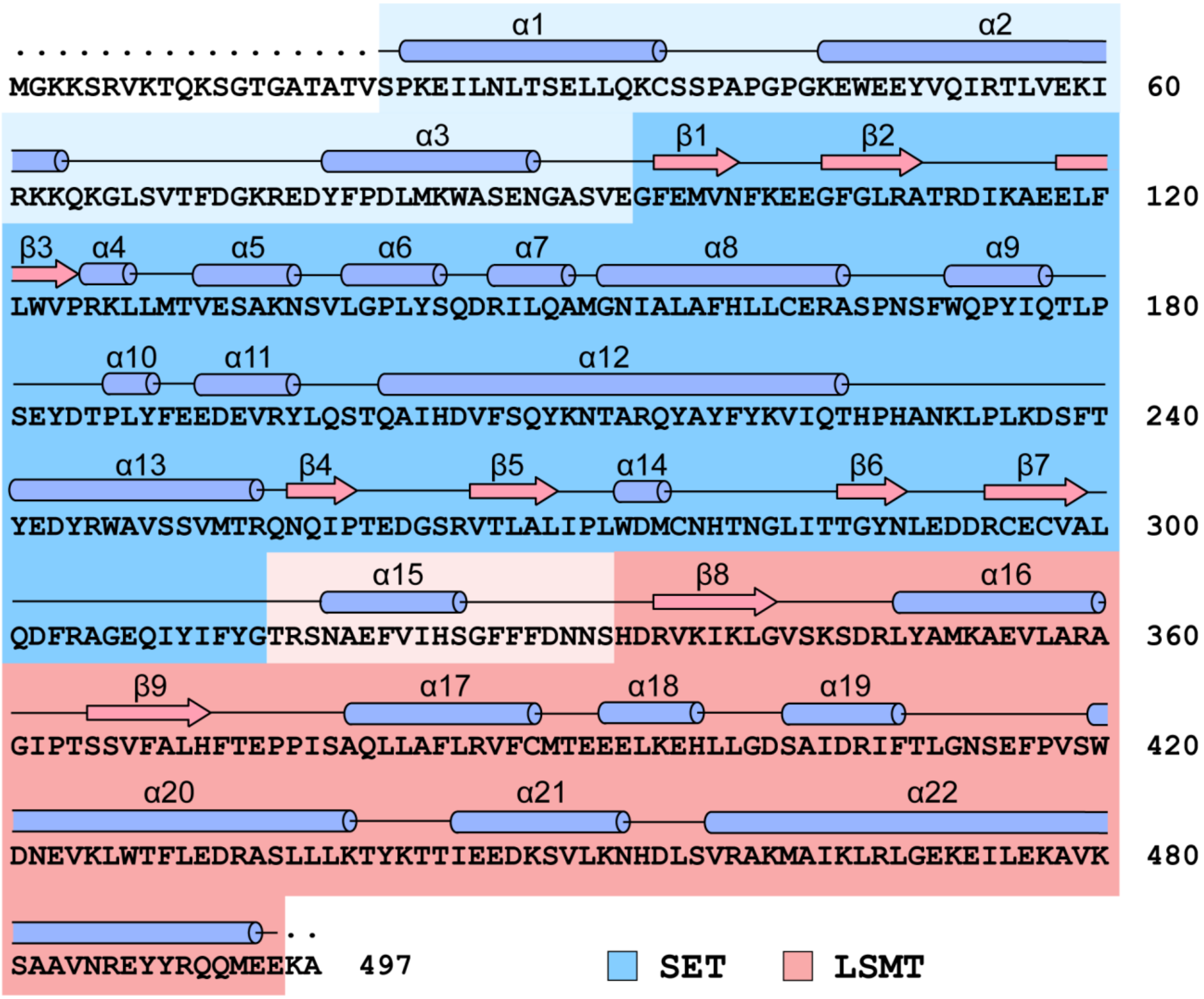

Supplementary Fig. S1 Structural based secondary structure assignment.

α helices and β strands are colored blue and salmon, respectively. SET domain and Rubisco LSMT binding domain are shaded by the denoted box color. Invisible residues in the structure are denoted by dots.

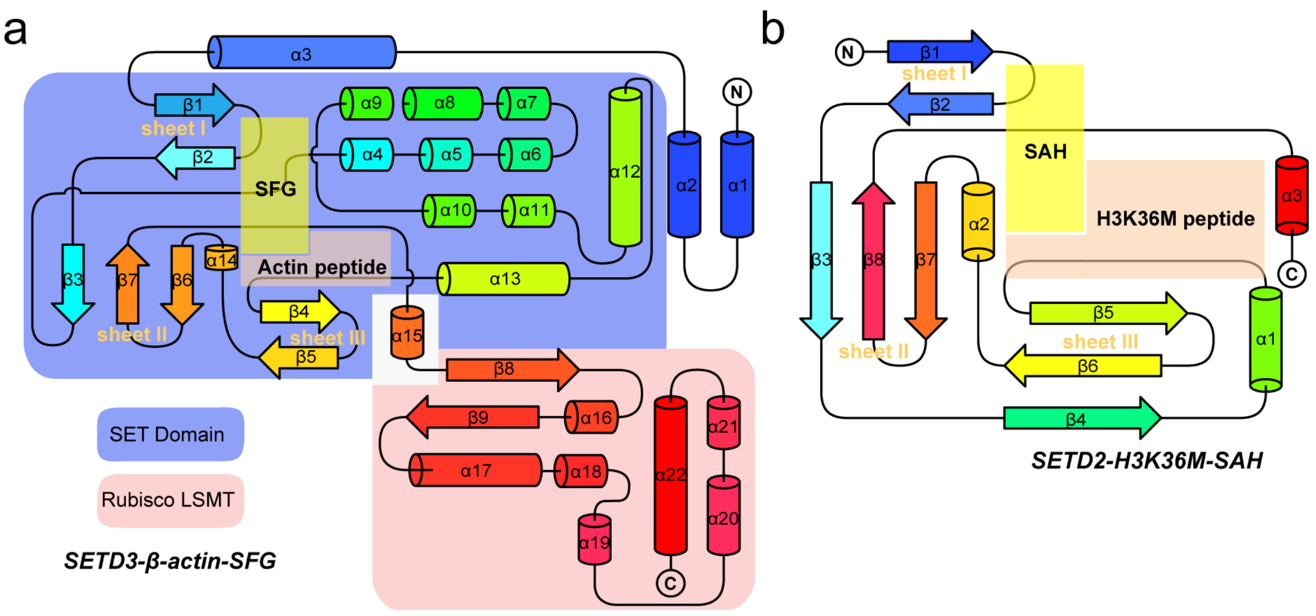

Supplementary Fig. S2 Topology of SETD3 and SED2.

a Topology diagram of ternary complex SETD3$_{20\text{-}494}$-Actin-SFG. α helices and β strands are numbered and rainbow-colored from the N to C terminus. SET domain and Rubisco LSMT binding domain are shaded blue and pink, respectively. Actin peptide- and SFG-binding sites are denoted as light pink and yellow boxes, respectively.

b Topology diagram of ternary complex SETD2-H3K36M-SAH. α helices and β strands are numbered and rainbow-colored from the N to C terminus. H3K36M peptide- and SAH-binding sites are shaded light pink and yellow, respectively.

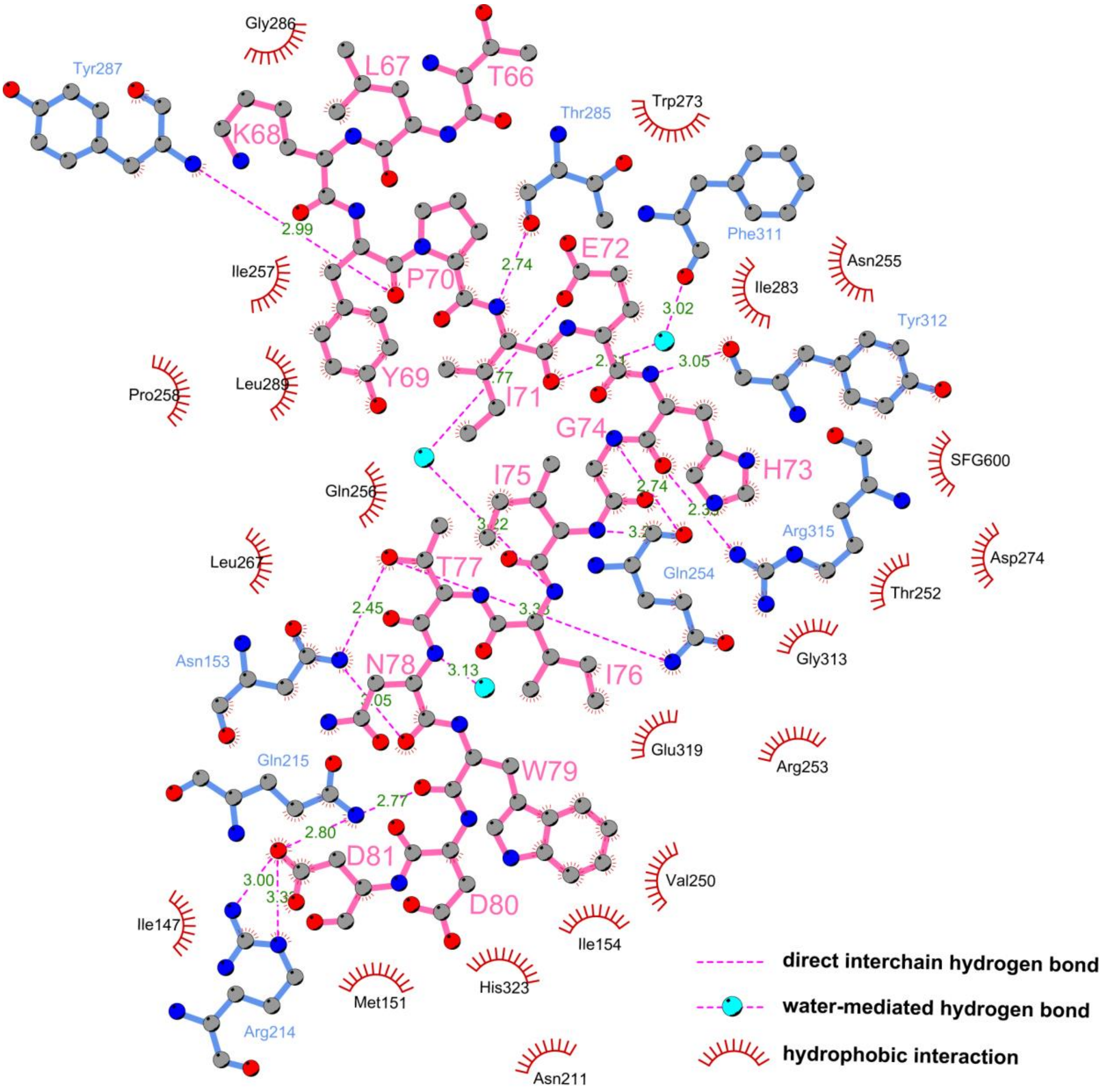

Supplementary Fig. S3 Binding details between SETD3 and actin peptide.

LIGPLOT diagram listing critical interactions between the actin peptide (66-81) and SETD3. Actin peptide (pink) and key residues of SETD3 (light blue) are depicted in a *ball-and-stick* model. *Gray sphere*, carbon; *Blue sphere*, nitrogen; *red sphere*, oxygen; *cyan sphere*, water molecule.

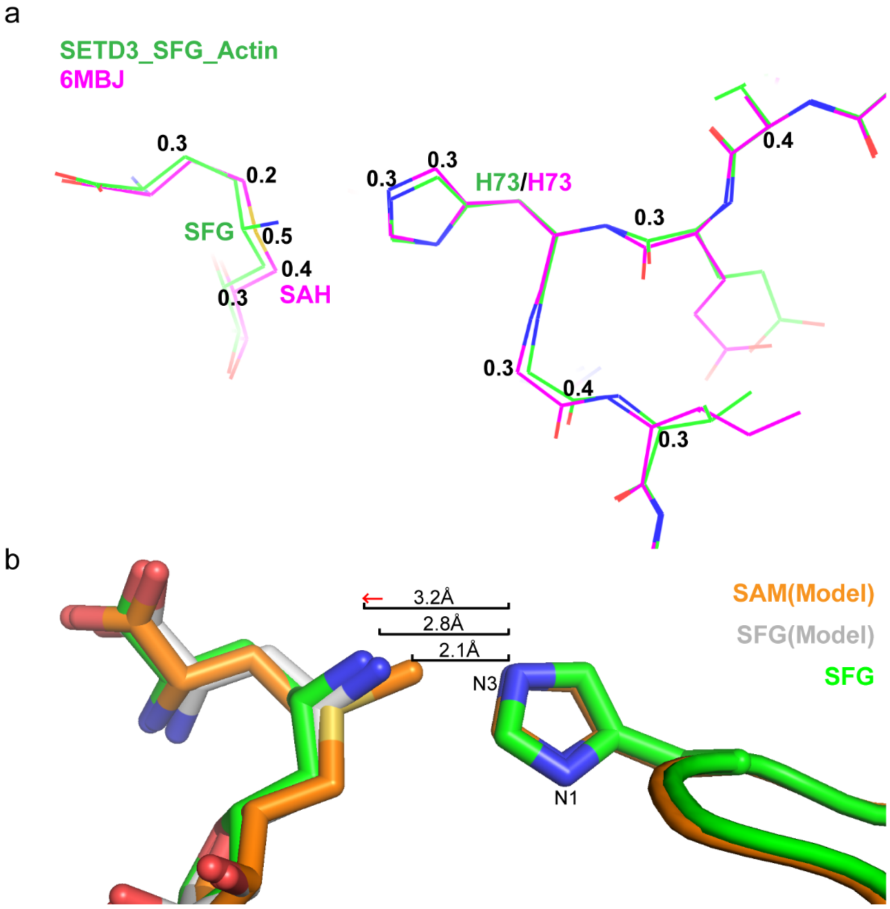

Supplementary Fig. S4 Steric constraints introduced by SFG/SAM binding

a Structural superposition of the “SETD3-SFG-actin” ternary complex (green) and “SETD3-SAH-actin” ternary complex (purple, PDB code: 6MBJ). Numbers are measured distances between the counterpart atoms of the two complexes in the unit of Å. Note the global outer shift of both SFG and actin peptide in the SETD3-SFG-actin complex.

b Structural superposition of SAM model, SFG model and SFG based on 6MBJ. Numbers are measured distances between N3 atom of histidine and CH_3 in SAM, or NH_2 in SFG(model and real structure). The red arrow shows outer shift of SFG between model and real structure.

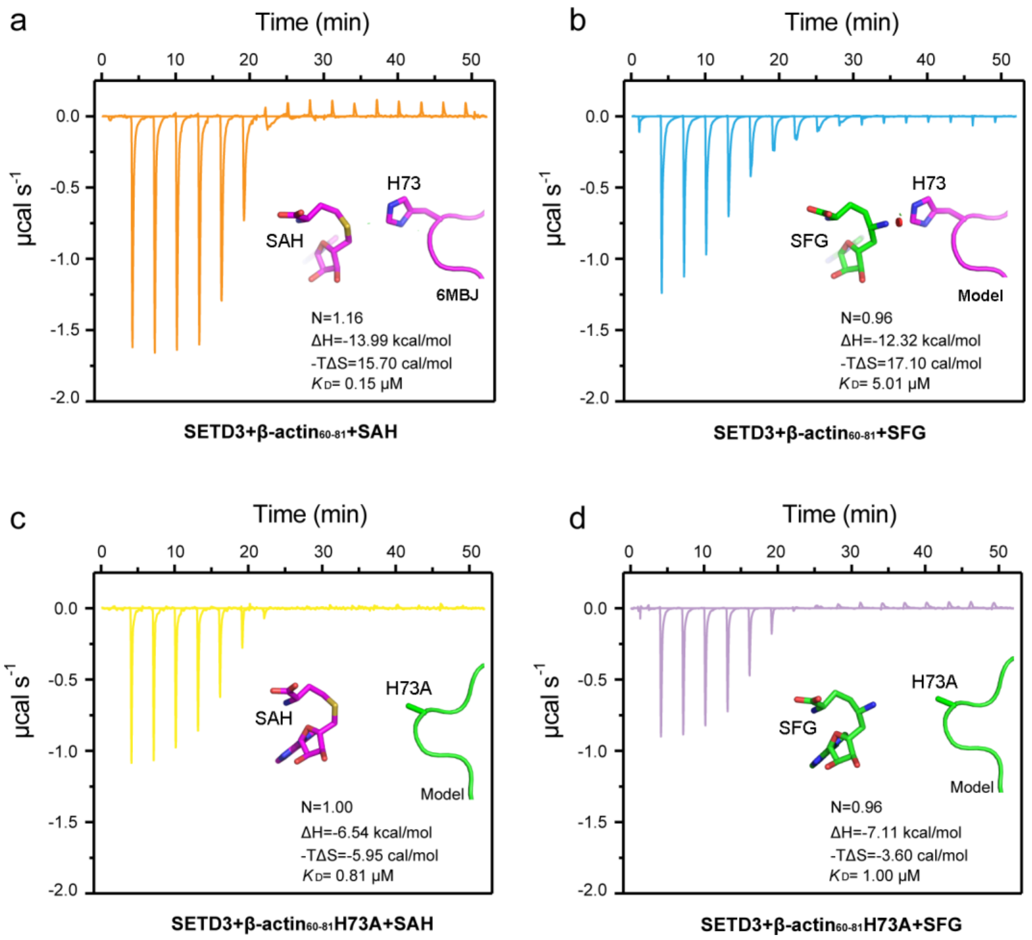

Supplementary Fig. S5 ITC titration curves of SETD3 titrated with wild type β-actin peptide (a, b) or H73A mutant peptide (c, d) in the presence of SAH or SFG. All the models were generated based on the SETD3-SAH-actin ternary complex (PDB: 6MBJ). Red and green discs shown in panel b denote steric clashes.
